# DMF-Activated Nrf2 Ameliorates Palmitic Acid Toxicity While Potentiates Ferroptosis Mediated Cell Death: Protective Role of the NO-Donor S-Nitroso-N-Acetylcysteine

**DOI:** 10.3390/antiox12020512

**Published:** 2023-02-17

**Authors:** Diana Abu-Halaka, Adi Shpaizer, Haim Zeigerman, Joseph Kanner, Oren Tirosh

**Affiliations:** Institute of Biochemistry, School of Nutritional Sciences, Food Science and Nutrition, The Robert H. Smith Faculty of Agriculture, Food and Environment, The Hebrew University of Jerusalem, Rehovot 76100, Israel

**Keywords:** lipotoxicity, ferroptosis, nitric oxide donors, Nrf2

## Abstract

Nonalcoholic fatty liver disease (NAFLD) is the most prevalent chronic liver disease that can develop into an aggressive form called nonalcoholic steatohepatitis (NASH), which ultimately progresses to cirrhosis, hepatocellular carcinoma (HCC), and end-stage liver failure. Currently, the deterioration of NAFLD is attributed to specific lipid toxicity which could be due to lipotoxicity and/or ferroptosis. In the current study, we evaluated the involvement of the nuclear factor erythroid 2 (NFE2)-related factor 2 (Nrf-2), which is a main activator of phase II metabolism in the two types of lipid-induced toxicity in hepatocytes, lipotoxicity by saturated fatty acids, and in ferroptosis, and the effect of NO donor treatment. AML12 cells were exposed to 600 μM palmitic acid to induce lipotoxicity or treated with 20 μM erastin or 5 μM RSL3 for ferroptosis. In SFA-lipotoxicity, pretreatment with the Nrf2 activator dimethyl fumarate (DMF) managed to ameliorate the cells and the oxidative stress level while aggravating ferroptosis due to emptying the thiol pool. On the other hand, the nitric oxide (NO)-donor, S-nitroso-N-acetylcysteine (NAC-SNO) proved to be effective in the prevention of hepatocytes ferroptosis.

## 1. Introduction

Nonalcoholic fatty liver disease (NAFLD) comprises a spectrum of hepatic pathology, from simple steatosis (SS) to nonalcoholic steatohepatitis (NASH) and cirrhosis [1,2], and is an increasingly important global public health problem.

It has been shown that the deterioration from simple steatosis to NASH is by the combination of free fatty acid accumulation in hepatocytes alongside the adaptation of the cells to toxic lipids and their derived metabolites, such as saturated fatty acids (SFA), lipid peroxidation products and others [3,4]. All of these activate mitochondrial dysfunction, inflammatory pathways, and oxidative stress which lead to cellular damage and cell death, a phenomenon known as lipotoxicity [5,6,7]. Lipotoxicity is facilitated via overnutrition, increased lipolysis, enhanced adipocytes apoptosis, and impaired adipogenesis, which together lead to a reduced capacity of the adipose tissue to store triglycerides and thus led to their accumulation in visceral fat and liver [8]. Overall, lipotoxicity is specially induced by palmitic acid (16:0) and stearic acid (18:0), the most abundant saturated fatty acids in the diet and circulation [9].

Another form of programmed cell death that is related to lipid toxicity is ferroptosis. This form of cell death is gaining more attention in the context of NAFLD. Unlike apoptosis, ferroptosis is an iron-dependent cell death that occurs through the lethal accumulation of lipid-based reactive oxygen species (ROS) when glutathione (GSH)-dependent lipid peroxide repair systems are compromised [10,11,12,13]. Several studies confirmed the crucial role of cellular cysteine deprivation and glutathione depletion in inducing ferroptotic cell death and demonstrated that both iron chelators and lipophilic antioxidants could block such death from occurring [14,15,16,17]. 

Nitric oxide (NO) donors’ anti-ferroptotic activity under various ferroptotic conditions was previously established [18]. S-nitroso-N-acetylcysteine (NAC-SNO) is a NO donor, that is synthesized from the thiol N-acetylcysteine (NAC) and was shown to be an antioxidant [19]. We conducted several studies on the effects of NO and NO-compounds on lipid peroxidation in model systems and foods. The results demonstrated that NO, nitric oxide myoglobin (Mb-NO), nitric oxide ferrous complexes (X-Fe-NO), and S-nitroso-cysteine (cys-SNO) act directly as antioxidants [20,21,22]. All of these compounds, during dissociation, in part-release nitric oxide (NO), found to act as an antioxidant, by direct inhibition of the Fenton reaction, generation of oxoferryl ions [23], and inhibition of lipid peroxidation by lipoxygenase, cyclooxygenase, hemoglobin, and myoglobin [24]. Thus, it may prevent cell death while also improving the levels of thiols or GSH in cells.

In the current research, we aimed to elucidate the intricate relationship between the activation of the antioxidant defense system of the nuclear factor erythroid-derived-2-like 2 (Nrf2)-Kelch-like ECH-associated protein 1 (Keap1)-antioxidant response element (ARE) pathway and ferroptosis or lipotoxicity in hepatocytes. Hence we have used Dimethyl fumarate (DMF), an FDA-approved oral drug [25] that has antioxidant properties as an electrophile potentiating the Nrf2 antioxidant pathway [26,27]. 

Herein, we studied the role of Nrf2 in the two different death types, SFA-induced death and ferroptosis by cystine deprivation or GPX4 inhibition. In addition, we evaluated which type of cell death in hepatocytes is sensitive to NO treatment.

## 2. Materials and Methods

### 2.1. Chemicals

Sodium nitrite, N-acetylcysteine (NAC), dimethyl fumarate (DMF), palmitic acid (PA), 1S,3R-RSL 3 (RSL3), 3-(4,5-dimethylthiazol-2-yl)-2,5-diphenyltetrazolium bromide (MTT) reagent, erastin, free fatty acid and bovine serum albumin (BSA) were purchased from Sigma-Aldrich. 5-Chloromethylfluorescein diacetate (Green CMFDA) was purchased from Cayman. DMF, RSL3, erastin, and Green CMFDA were dissolved in dimethyl sulfoxide (DMSO).

A palmitate acid–BSA conjugate was prepared as described previously [28]. Briefly, the palmitic acid—BSA stock solution was prepared by coupling free fatty acids with bovine serum albumin (BSA). First, palmitate was dissolved in pure ethanol at a concentration of 195 mM. This FFA stock solution was then added to a prewarmed 10% *w*/*w* BSA solution (37 °C) to achieve a final FFA concentration of 3 mM, and this solution was allowed to incubate in a water bath for an additional 10 min. All vehicle treatments were prepared using stocks of 10% *w*/*w* BSA with an equivalent volume of ethanol added to match the concentration in FFA stocks.

### 2.2. Cell Line

Mouse hepatocytes AML12 cells (CRL-2254™-ATCC), stably transfected with human transforming growth factor α and with typical hepatocytes features, were used in this study. AML12 cells were maintained in DMEM supplemented with 10% (*v*/*v*) fetal bovine serum (FBS) and 1% (*v*/*v*) penicillin-streptomycin. The growth medium was replaced every 2–3 days. Cells were cultured in 5% CO_2_ in a humidified atmosphere at 37 °C.

### 2.3. Preparation of S-Nitrosothiols (RSNO)

S-nitrosothiols were synthesized as previously described [29] by reacting equimolar amounts of sodium nitrite and the corresponding thiol in HCl (1 M) for 5 min at room temperature.

### 2.4. Propidium Iodide Cell Viability Assay 

Following treatment, cells were trypsinized, washed with phosphate-buffered saline (PBS), and centrifuged for 5 min for 2740× *g* (Thermo Scientific™, Heraeus Megafuge 16R, Osterode am Harz, Germany) at 4 °C. Then, cells were resuspended in PBS. Cells were filtered through a 90-µm mesh grid and stained with PI (2 µg/mL). Data were collected from 10,000 cells, measured by flow cytometry (FACSCalibur, Becton–Dickinson, Franklin Lakes, NJ, USA) with the fluorescence setting of excitation at 488 nm and emission at 575 nm, and analyzed using CellQuest Version 3.3 software.

### 2.5. MTT Cell Viability Assay

AML12 cells (7.5 × 10^5^ cells/mL) were seeded in 96-well plates. For cell viability detection, 10 μL 3-(4,5-dimethylthiazol-2-yl)-2,5diphenyltetrazolium bromide (MTT) reagent (5 mg/mL) in phenol red-free DMEM was added to cells. After being incubated at 37 °C for 3 h, the supernatant of the cell culture was discarded, and the visible formazan crystals were dissolved by 100 μL DMSO. Then the values at an absorbance of 560 nm were measured and recorded using a Tecan infinite 200 PRO microplate reader (Tecan Trading AG, Männedorf, Switzerland).

### 2.6. DNA Fragmentation Assay

Following treatment, cells were trypsinized, washed with PBS, and fixed in 0.5 mL of 1% (*v*/*v*) paraformaldehyde (in PBS), on ice for 30 min. Then, cells were centrifuged for 5 min at 2740× *g* (Thermo Scientific™, Heraeus Megafuje 16R) and the supernatant was discarded. Cells were suspended in DNAF solution (0.05 mg/mL PI, 0.1% (*v*/*v*) Triton x-100, 0.1% (*w*/*v*) sodium citrate dihydrate). Data were collected from 10,000 cells, fluorescence was measured by flow cytometry (FACSort, BD) with excitation at 488 nm and emission at 530 nm and we analyzed these using CellQuest Version 3.3 software. 

### 2.7. Gene Expression 

Total RNA was isolated using the Tri Reagent (Sigma–Aldrich, Rehovot, Israel) solution. One microgram of total RNA was converted into cDNA. Real-time PCR was performed using the 7300 Real-Time PCR system (Applied Biosystems, Singapore) and carried out with the PerfeCTa SYBR Green FastMix (Quanta bio, Gaithersburg, MD, USA). 18S was used as the control gene for real-time PCR. The sequences of the primers used are appear in Table 1. 

### 2.8. Nuclear Extracts Preparation

Nuclear extracts were prepared using two extraction buffers. In brief, treated cells were washed twice with cold PBS and covered with cold hypotonic buffer (10 mM HEPES, pH 7.0, 10 mM KCl, 3 mM MgCl_2_, 1 mM dithiothreitol (DTT), 0.05% NP-40, 1 mM EDTA, 10 mM NaF, 0.1 mM Na_3_VO_4_, 1 mM phenylmethylsulfonyl fluoride (PMSF), 1% protease inhibitor cocktail), scraped and incubated for 10 min on ice. Then, the samples were centrifugated at 500× *g* for 5 min. The supernatant containing the cytoplasmic extracts was collected and frozen at −80 °C. The pellets were resuspended in cold hypertonic buffer (50 mM HEPES, pH 7.0, 420 mM NaCl, 1.5 mM MgCl_2_, 1 mM dithiothreitol (DTT), 1% NP-40, 5% glycerol, 0.1 mM EDTA, 10mM NaF, 0.1 mM Na_3_VO_4_, 1 mM phenylmethylsulfonyl fluoride (PMSF), 1% protease inhibitor cocktail). The samples were frozen and thawed three times and were incubated on ice for 30 min, then centrifuged at 17,000× *g* for 10 min at 4 °C. The supernatant (containing the nuclear extract) was collected and frozen at −80 °C. The protein concentration from the cytosolic and nuclear fraction was evaluated using the Bradford method.

### 2.9. Western Blot Analysis

Protein content was measured using the BCA method. (34) Proteins (20 μg) were separated on 10% SDS-polyacrylamide gel electrophoresis and transferred to nitrocellulose membranes. Membranes were blocked with 5% BSA in TBST (Tris buffer NaCl-Tween) at room temperature for 1 h. After blocking, the membranes were incubated with first antibodies: Anti-Nrf2 (Abcam, ab137550, 1:1000), Anti-TATA binding protein TBP antibody [1TBP18] (Abcam, ab818, 1:2000) and Anti-GAPDH (Cell signaling, 14C10, 1:10,000) overnight at 4 °C. The membranes were then washed with TBST three times for 15 min, and secondary antibodies (1:10,000) peroxidase-conjugated goat anti-mouse IgG (Jackson, 115-035-003) for TBP or peroxidase-conjugated Goat Anti-rabbit IgG (Jackson, 111-035-144) for Nrf-2 and GAPDH were used to detect specific antibody bindings for 1 h at room temperature. The resulting band intensity was analyzed on an Image Gel pro system (Bio-Rad). Cytosolic and nuclear Nrf-2 bands intensity were normalized to GAPDH and TBP bands intensity respectively.

### 2.10. Lipid Peroxidation Analysis

Lipid peroxidation was measured using a TBARS (thiobarbituric acid reactive substances) assay. AML12 cells were trypsinized, washed with PBS, and centrifuged for 5 min for 2740× *g* at 4 °C. The pellet was dissolved with 0.5 mL of 12% (*v*/*v*) TCA (trichloroacetic acid) and centrifuged for 5 min. 400 µL of the supernatant was separated and mixed with 250 µL of 20 mM TBA (thiobarbituric acid) in 50% (*v*/*v*) acetic acid. The samples were incubated for 60 min at 100 °C. 30 μL of the sample was injected and separated with a C-18 Phenomenex column, model RP-18, and detected with an HPLC (Merck/Hitachi HPLC system, LaChrom L 7100 pump, and LaChrom Fluorescence Varian ProStar 363 detector) set at 532 nm excitation and 553 nm emission. The mobile phase consisted of a 35: 65 (*v*/*v*) mixture of methanol and 0.05 M potassium phosphate buffer, pH 7, and the flow rate was 0.6 mL min^−1^. MDA standard solutions (produced by Tetraethoxypropane) were used to generate a standard curve. MDA concentrations were normalized by protein quantity measured by Bradford assay.

### 2.11. Cellular Thiol Levels Analysis 

Cellular thiol levels were assessed using 5-Chloromethylfluorescein diacetate (Green CMFDA) with flow cytometry. Cells were washed with PBS and incubated at 37 °C for 15 min with 4 µM CMFDA in FCS-free DMEM. Then the CMFDA medium was replaced with FCS-free DMEM. Thirty minutes later, the cells were trypsinized, centrifuged at 685× *g*, for 5 min at 4 °C, and re-suspended in PBS. Cells were kept on ice until fluorescence was measured by flow cytometry. Fluorescent emission was measured at 530 nm with excitation set at 488 nm. Data were collected from 10,000 cells for each of the 4 replicates per treatment.

### 2.12. Statistical Analysis

Values are presented as means ± SD. Data were analyzed by analysis of variance (one-way ANOVA) followed by the Tukey-Kramer HSD post-hoc test utilizing the JMP 16 Pro software suites (SAS Institute, Cary, NC, USA). The significance level was *p* < 0.05 for all analyses. 

## 3. Results 

### 3.1. DMF Worsens Ferroptosis while Ameliorates Lipotoxicity in Hepatocytes

**Figure 1:**
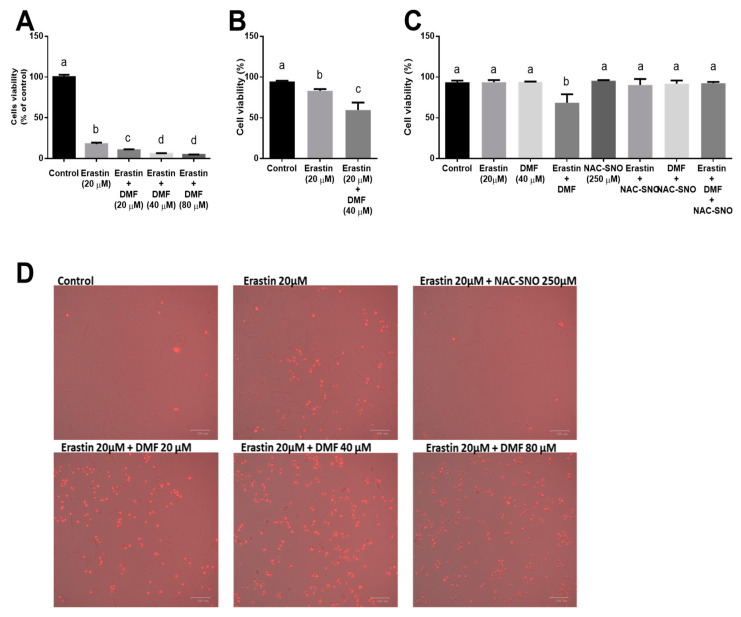
**The effect of DMF on cell viability in ferroptosis**. (**A**) Cell viability was evaluated by MTT assay in AML12 treated with 20 µM erastin with or without 20, 40, and 80 µM DMF for 24 h. (**B**) Cell death was measured by propidium iodide (PI) staining qualitatively by flow cytometry of AML12 cells treated with 20 µM erastin with or without 40 µM DMF and 250 µM NAC-SNO for 24 h. (10,000 cells, n = 3) (**C**) Cell death measured by propidium iodide (PI) staining qualitatively by flow cytometry of AML12 treated with erastin with or without DMF (10,000 cells, n = 3). (**D**) Propidium iodide (PI) staining of AML12 treated with erastin, with DMF and NAC-SNO. Red-stained cells: dead/damaged cells; non-stained cells: normal cells. Means with different letters are statistically different (*p* < 0.05).

To induce ferroptosis, we treated AML12 hepatocytes with erastin, an inhibitor of the cystine-glutamate antiporter system X_c_^−^, for 24 h. Cell viability was measured by MTT assay. 20 µM of erastin dramatically reduced cell vitality. Erastin treatment in combination with DMF resulted in the exacerbation of the toxic effect of erastin by 2-folds and more in DMF dose-dependent manner (Figure 1A) as measured by the MTT assay. In addition, staining with propidium iodide (PI) showed that DMF further potentiates the lethality of erastin (Figure 1B). It also indicated that treatment with the antioxidant and NO donor NAC-SNO protected the cells against the toxic effect of erastin in combination with DMF (Figure 1C). Images of PI staining in Figure 1D indicates that treatment with NAC-SNO protected the cells against the effect of erastin. In ferroptosis, the effect on membrane integrity loss as was evaluated by PI staining was less prominent (20–40% dead cells) as was evaluated by flow cytometer compared to the metabolic/death effect on the cells as was evaluated by the MTT assay. Therefore, we used the MTT assay in the ferroptotic model in later experiments.

**Figure 2:**
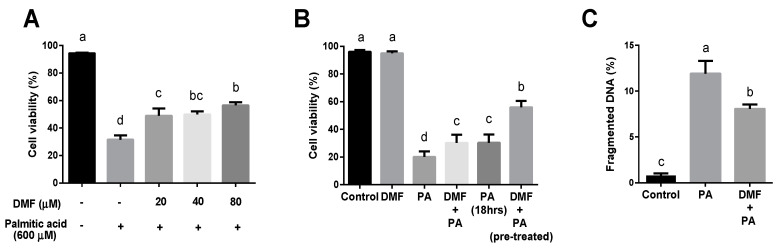
**The effect of DMF pretreatment on PA-induced lipotoxicity.** Cell death was measured by membrane permeability to PI using flow cytometry analysis in (**A**) AML12 treated with 600 µM palmitic acid (PA) and increasing concentrations of dimethyl fumarate (DMF) for 24 h (10,000 cells, n = 3). (**B**) AML12 treated with 40 µm DMF for 6 h before 0.6 mM PA (pretreated) or simultaneously for 24 h (10,000 cells, n = 3). (**C**) DNA fragmentation was evaluated using Flow cytometry analysis in AML12 treated with 40 µM DMF for 6 h before 600 µM PA. Means with different letters are statistically different (*p* < 0.05).

Additionally, we examined the effect of DMF on SFA-induced death. AML12 cells were treated with increasing doses of DMF and 0.6 mM palmitic acid (PA) for 24 h. Hepatotoxicity was assayed by flow cytometry, utilizing PI staining as an indicator for cell death. In accordance with previously conducted studies, palmitic acid-induced significant toxicity in AML12 cells, and DMF was found to protect and decrease cell death (Figure 2A). Remarkably, 6 h of pretreatment with DMF, before palmitic acid, showed a more considerable decrease in cell death rather than the DMF-PA concomitantly treatment for 24 h (Figure 2B, Figure S1 in Supplementary Materials). Additionally, DMF pre-treated hepatocytes showed a notable decrease in the extent of the fragmented DNA (Figure 2C). Therefore, to reach maximal protection of DMF, we used 40 µM DMF pretreatment for 6 h in the following tests. Surprisingly, NAC-SNO had no protective effect in this model of PA-induced lipotoxicity in hepatocytes (data not shown).

### 3.2. NAC-SNO Prevents Cell Death in Ferroptosis

**Figure 3:**
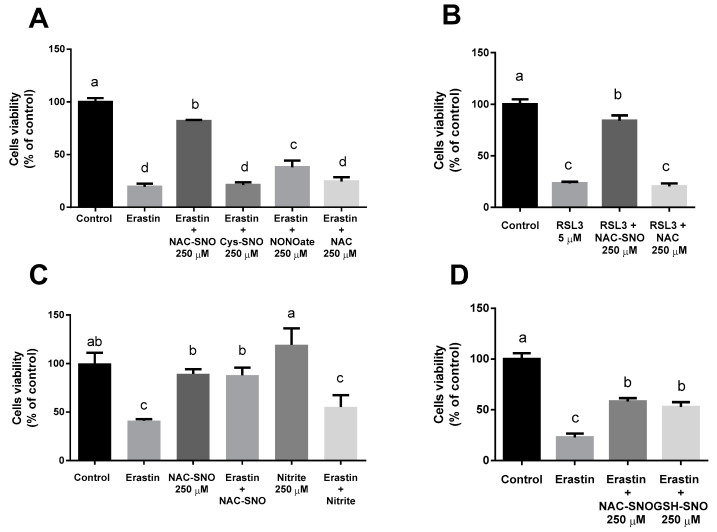
**The effect of NO donors on cell viability in ferroptosis.** Cell viability was measured by MTT assay of AML12 cells treated with (**A**) 20 µM Erastin and 250 µM NAC-SNO, S-nitroso-cysteine (cys-SNO), NONOate, and NAC for 24 h. (**B**) 5 µM RSL3 and 250 µM NAC-SNO and NAC for 6 h. (**C**) 20 µM Erastin with or without 250 µM nitrite and S-nitroso-N-acetylcysteine (NAC-SNO) for 24 h. (**D**) 20 µM Erastin and 250 µM NAC-SNO and S-nitroso-glutathione (SNO-GSH). Means with different letters are statistically different (*p* < 0.05).

To measure ferroptosis in vitro, we treated AML12 with two ferroptosis inducers: erastin, an inhibitor of system X_c_^−^, and RSL3, an inhibitor of glutathione peroxidase 4 (GPX4). Again, erastin reduced cell vitality after 24 h while supplementation of 250 µM S-nitroso-N-acetylcysteine (NAC-SNO) succeeded in preventing cell death, however, nitrite at the same concentration was ineffective in protecting the cells (Figure 3C). 

NAC-SNO’s protective effect was compared to different NO-donors as well as with N-acetylcysteine (NAC), which is an antioxidant and a precursor for NAC-SNO. We examined the ability of NAC-SNO, S-nitroso-cysteine (cys-SNO), diethylamine NONOate (DEA Nonoated), and S-nitrosoglutathione (GSH-SNO) to reduce cell death (Figure 3A,D). All supplements were at the same concentration (250 µM). The addition of NAC-SNO to erastin treatment showed almost 90% viability and DEA NONOate and GSH-SNO succeeded in reducing cell death to 50%. However, neither Cys-SNO nor NAC had a protective effect (Figure 3A). 

RSL3 also dramatically reduced cell viability similar to erastin. Cell viability was measured after 6h (Figure 3B). NAC-SNO significantly prevented cell death by RSL3 yet NAC failed to do so, indicating that for cell death prevention the capability to donate NO has greater importance than the capacity to supply thiols for the ferroptotic cell. 

### 3.3. DMF Lowers the Extent of Oxidative Stress in the Lipotoxicity Model but Increases It in Ferroptosis

**Figure 4:**
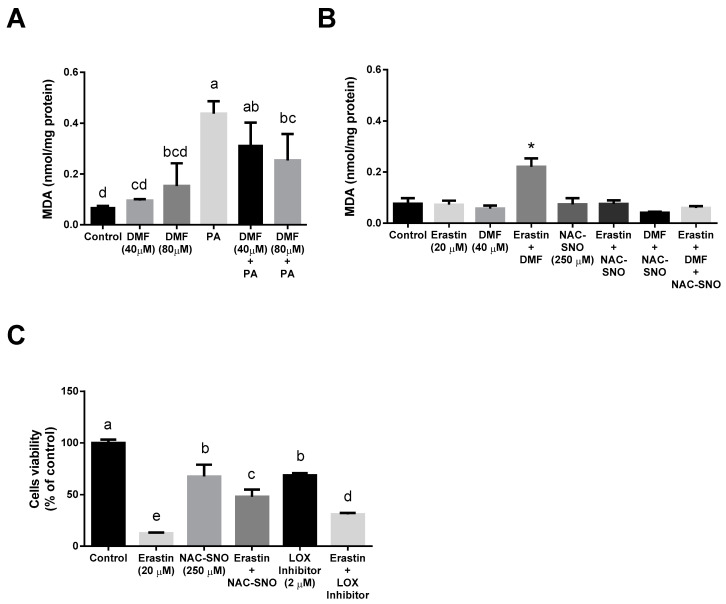
**Oxidative stress in lipotoxicity and ferroptosis:** MDA levels were measured in (**A**) AML12 treated with 40 or 80 µM DMF for 6 h before 600 µM palmitic acid (PA) for a total of 24 h. (**B**) AML12 cells were treated with 20 µM erastin with or without 40 µM DMF and 250 µM NAC-SNO for 6 h. (**C**) Cell viability was measured by MTT assay of AML12 treated with 20 µM Erastin with or without 250 µM NAC-SNO and 2 µM LOX inhibitor (ML351) for 24 h. Means with different letters are statistically different (*p* < 0.05). Column marked with * is statistically different from the rest (*p* < 0.05).

Malondialdehyde (MDA) was measured as a marker of oxidative stress. DMF pretreatment followed by exposure to palmitic acid tends to lower the levels of MDA (Figure 4A). In contrast to that, the erastin-DMF treatment led to a significantly elevated level of MDA (Figure 4B), whereas NAC-SNO was able to decrease it to control levels. ALOX12 (12-lipoxygenase) was shown to be related to ferroptosis. Oxidative stress causes the up-regulation of ALOX12 expression in vivo and in vitro [30,31]. We evaluated the effect of the selective LOX12/15 inhibitor ML351 on erastin-induced ferroptosis in AML12 cells using the MTT assay (Figure 4C). ML351 at 10 times the concentration of its IC_50_ had only a partial protective effect, indicating LOX independent ferroptosis pathways in hepatocytes which may be inhibited by NO donor treatment.

### 3.4. DMF Activates Nrf2-Related Genes in Both Lipotoxicity and Ferroptosis 

**Figure 5:**
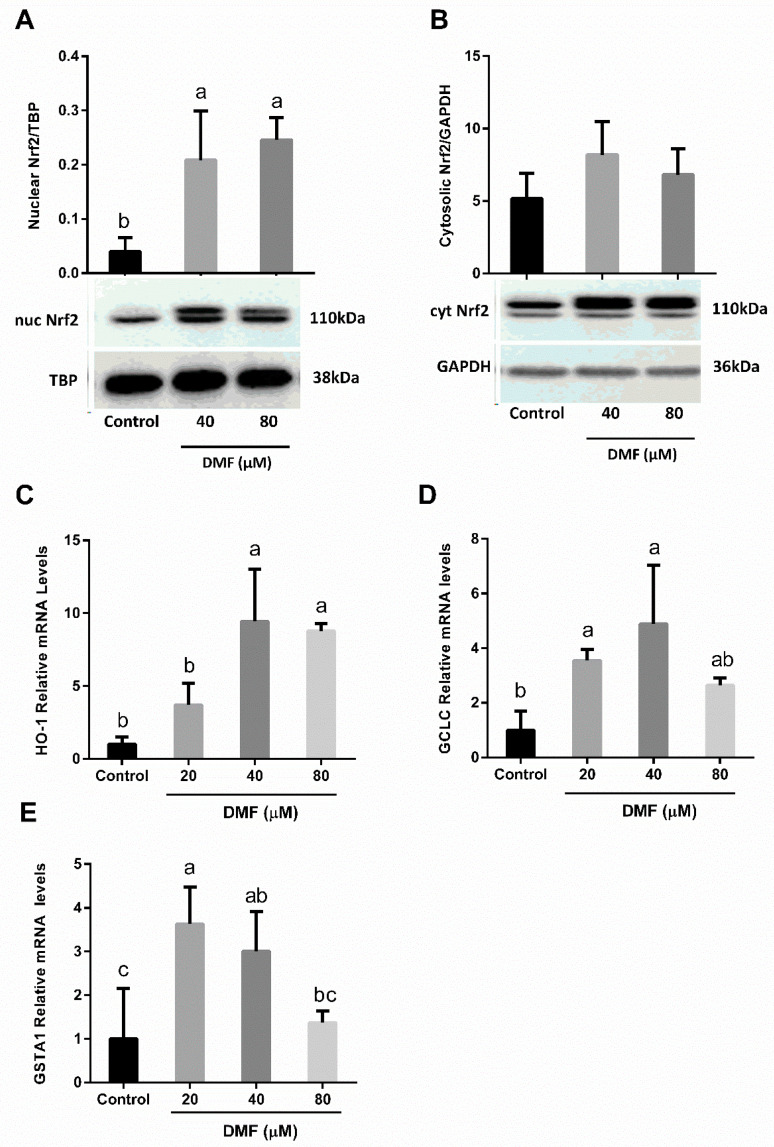
**The effect of DMF on Nrf2 activation.** (**A**,**B**) Nrf2 protein expression in AML12 hepatocytes treated with 40, or 80 μM DMF for 6 h and (**C**–**E**) Nrf2-related genes expression in AML12 hepatocytes treated with 20, 40, or 80 μM DMF for 6 h. Means with different letters are statistically different (*p* < 0.05).

**Figure 6:**
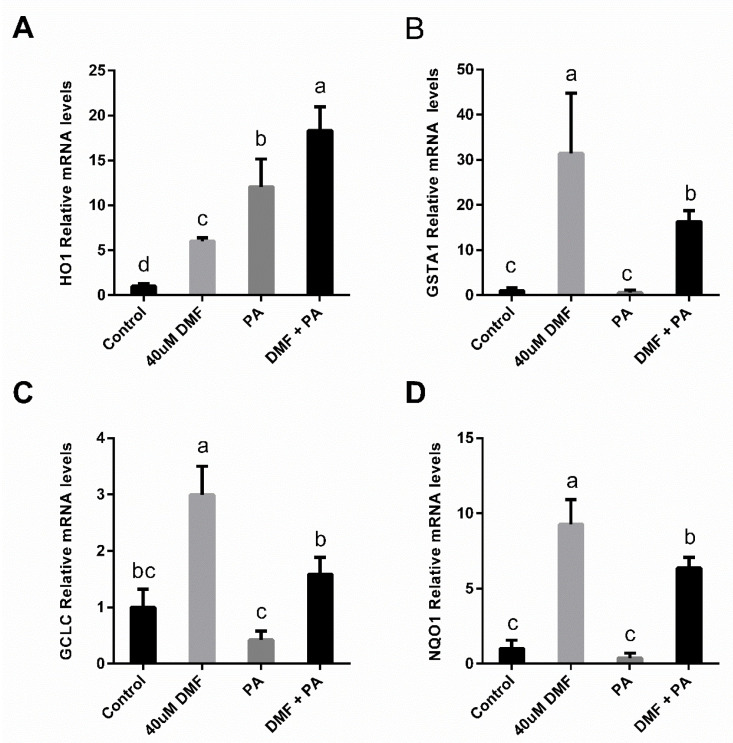
**The effect of DMF on Nrf2 activation in PA-induced lipotoxicity.** Nrf2-related genes expression (**A**–**D**) in AML12 hepatocytes treated with 40 μM DMF for 6 h followed by 0.6 mM PA for another 6 h. Means with different letters are statistically different (*p* < 0.05).

In order to validate that treatment with DMF for 6 h led to a strong Nrf2 activation we measured an increase in Nrf2 translocation to the nucleus and the alterations in Nrf2 target gene expression (Figure 5). According to the Western blot results for the Nrf2 (110 kDa), a double band was observed at 110 kDa in the DMF groups. Higher Nrf2 protein band intensity in the nuclear phase was found in the DMF groups when compared with the control group (Figure 5A,B). Consistently, analysis of gene expression showed that 40 µM DMF significantly induces the expression of Nrf2-related genes: heme oxygenase 1 (HO-1), glutamate-cysteine ligase catalytic subunit (GCLC), and glutathione s-transferase Alpha 1 (GSTA1) (Figure 5C–E). However, a higher dose of 80 µM DMF only slightly increased (not significantly) gene expression, although the translocation to the nucleus was evident. Nrf2-related genes were also induced as a result of the exposure to DMF-PA treatment (Figure 6A–D).

**Figure 7:**
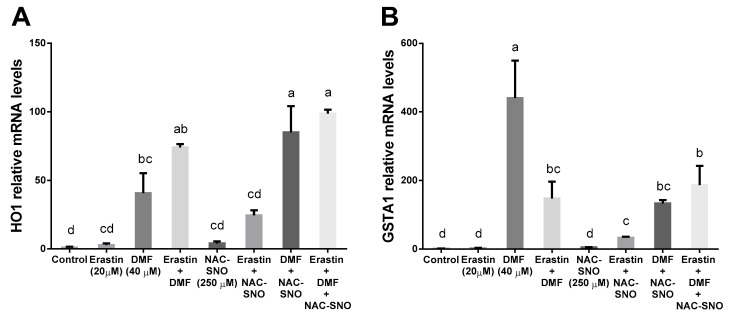
**The effect of DMF on Nrf2 activation in ferroptosis.** Nrf2-related genes expression (**A**,**B**) in AML12 hepatocytes treated with 20 μM erastin, 40 μM DMF, and 250 μM NAC-SNO for 6 h. Means with different letters are statistically different (*p* < 0.05).

DMF in combination with erastin and NAC-SNO increased Nrf2-related gene expression compared to untreated control. Both erastin and NAC-SNO increased HO-1 gene expression in cells treated with DMF compared to DMF alone (Figure 7A,B). On the other hand, erastin and NAC-SNO decreased GSTA1 gene expression in cells treated with DMF compared to DMF alone. The combination of erastin and NAC-SNO increased the expression of both genes compared to the control.

DMF in combination with erastin and NAC-SNO increased Nrf2-related genes expression compared to untreated control. Both erastin and NAC-SNO increased HO-1 gene expression in cells treated with DMF compared to DMF alone (Figure 7A,B). On the other hand, erastin and NAC-SNO decreased GSTA1 gene expression in cells treated with DMF compared to DMF alone. The combination of erastin and NAC-SNO increased the expression of both genes, compared to the control.

### 3.5. DMF Decreased Thiol Levels in Both Lipotoxicity and Ferroptosis

**Figure 8:**
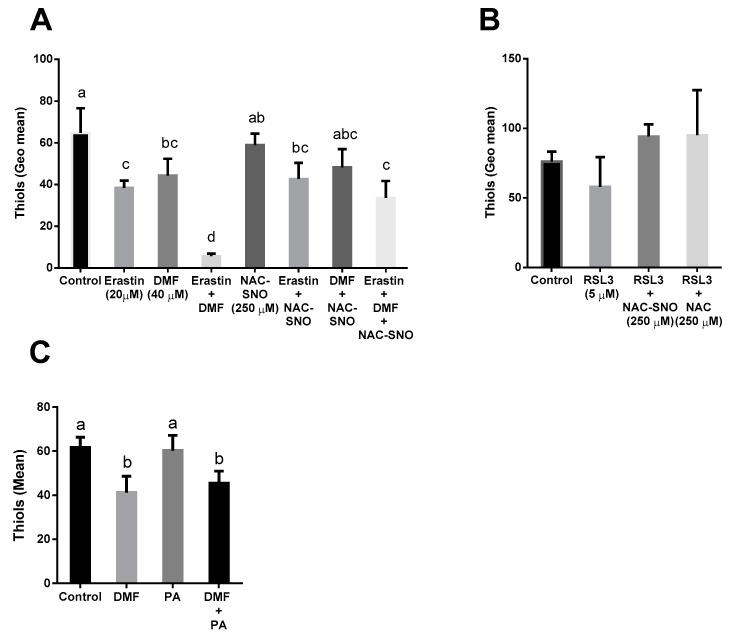
**Thiols in lipotoxicity and ferroptosis**. Thiols were measured with green CMFDA by FACS in AML12 treated with (**A**) Erastin (20 μM), DMF (40 μM), and NAC-SNO (250 μM) for 6 h. (**B**) RSL3 (5 μM), NAC-SNO (250 μM), and NAC (250 μM) for 6 h. (**C**) Pre-treated with DMF (40 μM) for 6 h before palmitic acid (PA) (600 µM) for another 6 h. Means with different letters are statistically different (*p* < 0.05).

Thiol levels were measured with 5-chloromethylfluorescein diacetate (CMFDA). Erastin inhibits system X_c_^−^, preventing cystine from entering the cells, therefore, lowering cysteine levels. Indeed, erastin alone significantly decreased thiol levels, however, thiol levels in cells treated with erastin in combination with DMF drastically decreased by 6-fold. NAC-SNO added with erastin and DMF managed to partly restore thiol levels (Figure 8A). RSL3 inhibits GPX4 thus limiting the oxidation of glutathione-to-glutathione disulfide. There was not a significant difference in thiol concentration in RSL3-treated cells compared to the control (Figure 8B). Treating AML12 with DMF alone or with PA resulted in a decline in thiol levels compared to control or PA (Figure 8C) indicating that activation of the Nrf2 system under conditions of lipotoxicity deplete thiol in hepatocytes to protect the cells (lower depletion compared to erastin-induced ferroptosis with DMF). 

## 4. Discussion

In the current study, we sought to measure the involvement of Nrf2 in two types of lipid-induced toxicity in hepatocytes: lipotoxicity due to saturated fatty acids, and ferroptosis.

To evaluate the effect of Nrf2 activation on cell death, DMF, an activator of Nrf2 was used while palmitic acid was employed as the toxic treatment. DMF ameliorates PA lipotoxicity. However, DMF pretreatment succeed in improving cell viability better than the concomitant treatment. DMF is an oral therapeutic agent for relapsing-remitting multiple sclerosis and psoriasis [25]. Recent studies have revealed the cellular protective and antioxidative effects of DMF in multiple sclerosis mouse models [32] and others disclosed the anti-apoptotic and anti-inflammatory contributions in liver ischemia-reperfusion injury (IRI) in rats [33]. In contrast, some studies, mainly in cancer cells, showed a pro-cell death effect of high doses of DMF due to impaired Nrf2 induction and transcriptional activities [34,35]. However, in this study, it was clear that the doses of DMF indeed activate the Nrf2 system. These doses also induce significant thiol depletion and lipid peroxidation to facilitate cell death. 

We have used ferroptosis as a model of cell death. In the ferroptotic hepatocytes, DMF exacerbates the toxic effect of erastin, indicating that activation of a defense system that promotes glutathione-dependent detoxification reactions will be less effective and even deleterious under conditions of limited thiol availability. In addition, activation of these defense systems will increase HO-1 expression under the control of Nrf2, the elevation of ferrous ions levels, and lipid peroxidation [36]. Free iron can increase ferroptosis and the progression of related diseases. 

However, in the lipotoxicity model, there is no limitation of thiol availability. Therefore, activation of high-impact transcription factors such as Nrf2 mitigates lipotoxicity. In ferroptosis, DMF elevates lipid peroxidation and drains the thiol pool. Glutathione detoxification of electrophiles (such as DMF) can deplete thiols both chemically and biologically, and further potentiate ferroptosis. On the other hand, NO derived nitrosylated cysteine molecules are beneficial in circumventing the depletion of thiols and may block Nrf2/HO-1 and free iron elevation to prevent hepatocyte ferroptosis.

To protect against ferroptosis, we used several NO donor molecules such as NAC-SNO and NAC, a known antioxidants. NAC-SNO but not NAC exerts protective effects against ferroptosis caused by erastin and RSL3. GSH-SNO and NONOate partly inhibit ferroptosis while nitrite and Cys-SNO did not protect the hepatocytes. As was previously demonstrated by us [29], Cys-SNO decomposes 40 times faster than NAC-SNO. In addition, S- to N-transnitrosation of Cys-SNO forms carbon-centered radicals and releases nitrogen instead of NO (at the expense of nitric oxide) thus making it a less effective antioxidant than NAC-SNO. Nitrite was found to be ineffective in protecting cells and to acting as an antioxidant. Nitrite releases NO as a result of heating, a reaction with transition metals in hemeproteins, or when exposed to low pH [18]. None of those conditions are achievable in the living cells as a way to prevent ferroptosis.

Oxidative stress plays a significant role in NAFLD initiation and progression and is caused by the excess of ROS generation compared to the antioxidant defenses, which leads to cell damage and death [37]. Researchers assume that iron overload may aggravate NAFLD and progress toward NASH by inducing inflammation and fibrosis [38]. Therefore, regulating the oxidative stress status may be one of the strategies to treat lipotoxicity and ferroptosis in NAFLD. Consistent with previous studies, our results show that exposure of hepatocytes to PA promotes ROS production [39] and leads to cellular apoptosis. Our data show that DMF’s protective effect in PA-induced ROS generation and toxicity is due to further activation of the Nrf2 pathway. PA toxic treatment-induced Nrf2 response indicating an adaptive response to the stress. PA by itself is not an electrophilic molecule; therefore, it most probably activates the Nrf2 pathway indirectly and in the presence of DMF, and there is also an additional direct activation effect. Regarding oxidative stress, the Nrf2 pathway is activated to regulate cellular redox and restores cell functions. PA probably activates the pathway by ROS generation [40] and DMF is an electrophile that activates the Nrf2 antioxidant response element (ARE) signaling pathway by covalent Keap1 modification and thus ameliorates lipotoxicity.

NO is found to be ineffective against PA-induced toxicity. SFA-induced toxicity is associated with oxidative stress [41]. In hepatocytes, palmitic acid at a toxic concentration increases the production of ROS and activates ER stress-related kinases, induces the apoptotic transcription factor C/EBP homologous protein (CHOP), and activates caspase 3, thus leading to apoptosis [42]. In contrast to lipotoxicity, our results indicate that nitric oxide donors are effective in preventing ferroptotic cell death in hepatocytes. NO, iron-NO complexes and S-nitroso compounds were found to act as antioxidants and inhibitors of lipoxygenase [24], which seems to act very significantly as an antioxidant in ferroptosis. Recently it was suggested that NO suppressed ferroptosis in macrophages via inhibition of hydroperoxyl-eicosatetraenoyl-phosphatidylethanolamine (HpETE-PE) production by 15-lipoxygenase (15-LOX) complexed with PE-binding protein 1 (PEBP1) [43]. However, it was reported that the pan-LOX inhibitor is effective in preventing ferroptosis in cells that possess and in cells that are devoid of lox activity, and that selective 5-lox inhibitors are ineffective in the prevention of ferroptosis [44]. Our data indicate that ML351, a cell penetrant, selective, and highly potent lipoxygenase-12/15 (15-Lipoxygenase-1, 12/15-LOX) inhibitor that exhibits protective effects against oxidative glutamate toxicity in mouse neuronal HT22 cells [45] had some limited effectiveness in preventing ferroptosis in mouse hepatocytes. Alox15, the gene encoding for 12/15-lipoxygenase (12/15-LOX), is markedly up-regulated in livers from apolipoprotein E-deficient (ApoE2/2) mice, which spontaneously develop nonalcoholic fatty liver disease [46]. However, considering that ALOX15 is expressed in macrophages, not hepatocytes [47] suggest that NO has more pathways to block ferroptosis in hepatocytes, which could also be related to the Nrf2 pathway. It is possible that NO can modulate Nrf2 to protect the liver following exposure to ferroptosis inducers. Previously, it was suggested that nitric oxide can suppress HO-1 expression both in vitro and in vivo [48]. NO can also inhibit the activation of nuclear factor-kB (NF-kB), within the HO-1 promoter to reduce HO-1 gene transcription [49].

## 5. Conclusions

The current understanding is that NAFLD can progress to NASH, fibrosis, fulminant hepatic failure, and HCC due to lipotoxicity and ferroptosis, a type of cell death that is related to lipid peroxidation. The current study elucidates the role of Nrf2 in lipid-induced hepatocyte damage and death and provides new insights into the pathophysiology of lipid toxicity and the understanding of the involvement of the Nrf2 and nitric oxide antioxidant defense system in regulating and preserving hepatocytes survival under steatotic stress.

## Figures and Tables

**Table 1:** Sequences of the primers used for quantitative real-time PCR.

Name	Accession Number	Reverse	Forward
18S	NR_003278.3	5′-CCTCAGTTCCGAAAACCAAC-3′	5′-ACCGCAGCTAGGAATAATGG-3′
HO-1	NM_010442.2	5′-CTTCCAGGGCCGTGTAGAT-3′	5′-CAGAAGGGTCAGGTGTCCA-3′
GCLC	NM_010295.2	5′-TCGCCTCCATTCAGTAACAA-3′	5′-CGAGGTGGAGTACATGTTGG-3′
GSTA1	NM_008181.3	5′-TGCAGCTTCACTGAATCTTGAAAG-3′	5′-CCCCTTTCCCTCTGCTGAAG-3′
NQO1	NM_008706.5	5′-CCTTTCAGAATGGCTGGCA-3′	5′-GGAAGCTGCAGACCTGGTGA-3′

## Data Availability

Data are contained within the article.

## Supplementary Materials

The following supporting information can be downloaded at: https://www.mdpi.com/article/10.3390/antiox12020512/s1, Figure S1: Light microscope live imaging and PI staining of AML12 pre-treated with DMF for 6 h before palmitic acid for a total of 24 h. Original images of Western blot membranes (related to Figure 5A,B).
