# Supplementary material for: DMF-Activated Nrf2 Ameliorates Palmitic Acid Toxicity While Potentiates Ferroptosis Mediated Cell Death: Protective Role of the NO-Donor S-Nitroso-N-Acetylcysteine
Source: Antioxidants (Basel). 2023 Feb 17;12(2):512. doi: 10.3390/antiox12020512 (PMC9952671; doi:10.3390/antiox12020512)
Supplement: Supplementary file 1 [file antioxidants-12-00512-s001.zip › antioxidants-2187907-supplementary.pdf]

Supplementary

To illustrate the effect of pre-treatment with DMF (quantified in figure 2B), hepatoxicity of 0.6 mM PA was also assayed by live imaging followed by PI staining in culture.

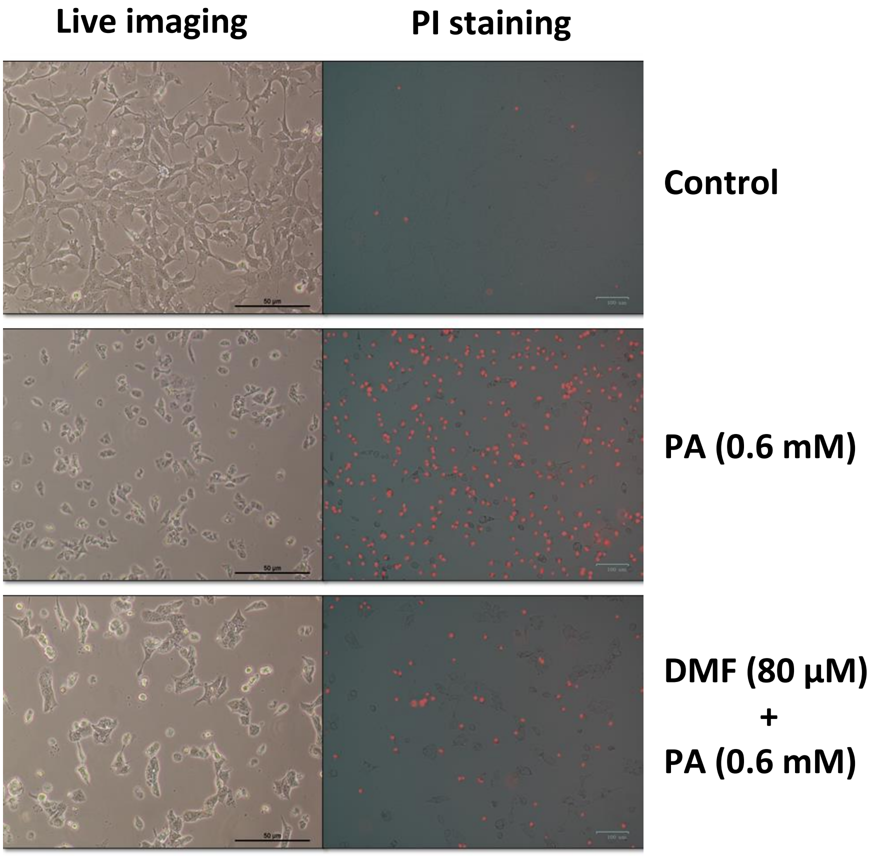

Figure S1: Light microscope live imaging of AML12 **(left)** and PI staining of AML12 **(right)** pre-treated with 80 µM DMF for 6 hours before 0.6 mM palmitic acid for a total of 24 hours. Red-stained cells: dead/damaged cells; non-stained cells: normal cells.

Original images of Western blot membranes (related to figure 5A, B)

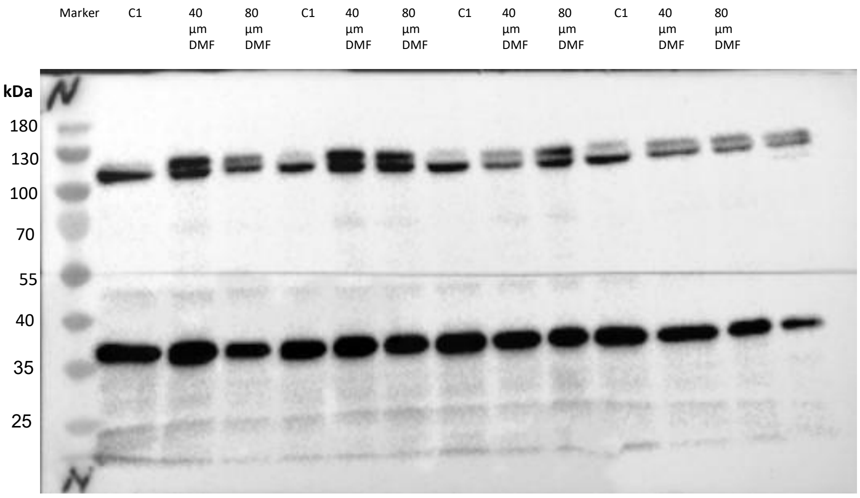

Membrane 1 (N): include only the nuclear phase

The upper lanes indicate the Nrf2, the lower lanes indicate TATA-binding protein (TBP) lanes 1,4,7,10: Control (DMSO)

Lane 2,5,8,11: 40 µm DMF

Lane 3,6,9,12: 80 µm DMF

Lane 13: sample contain equal amounts of the nuclear and the cytosolic fractions as a positive control

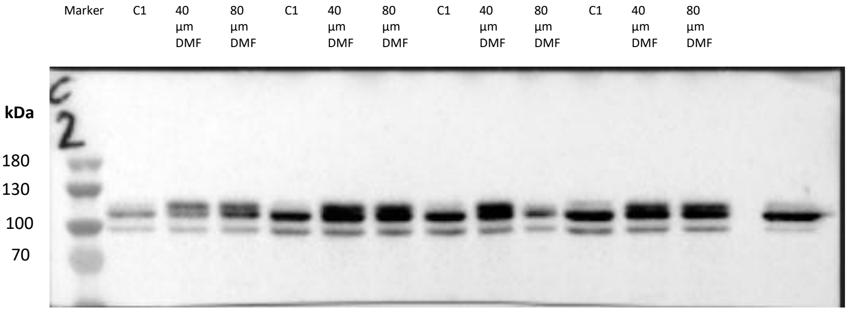

Membrane 2 (C) – upper part: include only the Cytosolic phase

The lanes indicate the Nrf2

lanes 1,4,7,10: Control (DMSO)

Lane 2,5,8,11: 40 µm DMF

Lane 3,6,9,12: 80 µm DMF

Lane 13: sample contain equal amounts of the nuclear and the cytosolic fractions as a positive control

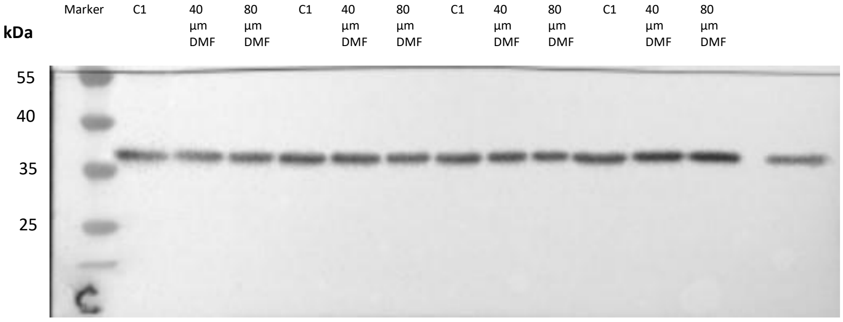

Membrane 2 (C) – lower part: include only the Cytosolic phase

The lanes indicate the GAPDH

lanes 1,4,7,10: Control (DMSO)

Lane 2,5,8,11: 40 µm DMF

Lane 3,6,9,12: 80 µm DMF

Lane 13: sample contain equal amounts of the nuclear and the cytosolic fractions as a positive control
